# Impact of a vegan diet on the human salivary microbiota

**DOI:** 10.1038/s41598-018-24207-3

**Published:** 2018-04-11

**Authors:** Tue H. Hansen, Timo Kern, Emilie G. Bak, Alireza Kashani, Kristine H. Allin, Trine Nielsen, Torben Hansen, Oluf Pedersen

**Affiliations:** 1 The Novo Nordisk Foundation Center for Basic Metabolic Research, Section of Metabolic Genetics, Faculty of Health and Medical Sciences University of Copenhagen, Copenhagen, Denmark; 2 Faculty of Health Sciences, University of Southern Denmark, Odense, Denmark

## Abstract

Little is known about the effect of long-term diet patterns on the composition and functional potential of the human salivary microbiota. In the present study, we sought to contribute to the ongoing elucidation of dietary effects on the oral microbial community by examining the diversity, composition and functional potential of the salivary microbiota in 160 healthy vegans and omnivores using 16S rRNA gene amplicon sequencing. We further sought to identify bacterial taxa in saliva associated with host inflammatory markers. We show that compositional differences in the salivary microbiota of vegans and omnivores is present at all taxonomic levels below phylum level and includes upper respiratory tract commensals (e.g. *Neisseria subflava*, *Haemophilus parainfluenzae*, and *Rothia mucilaginosa*) and species associated with periodontal disease (e.g. *Campylobacter rectus* and *Porphyromonas endodontalis*). Dietary intake of medium chain fatty acids, piscine mono- and polyunsaturated fatty acids, and dietary fibre was associated with bacterial diversity, community structure, as well as relative abundance of several species-level operational taxonomic units. Analysis of imputed genomic potential revealed several metabolic pathways differentially abundant in vegans and omnivores indicating possible effects of macro- and micro-nutrient intake. We also show that certain oral bacteria are associated with the systemic inflammatory state of the host.

## Introduction

Dietary effects on the human gut microbiota have been extensively studied, and long-term dietary habits have been shown to affect the diversity and composition of the human gut microbiota^1,2^. By comparison, studies exploring the microbiota of saliva are few and have, thus far, not revealed any substantial contribution of diet in shaping the oral microbial community. One study showed the salivary microbiota of traditional hunter-gatherers of the Batwa pygmy tribe in Uganda to have higher bacterial richness and profound differences in the abundance of 15 common bacterial genera compared to that of farmers from Sierra Leone and the Democratic Republic of Congo^3^. Noticeably, the pygmy diet is distinguished by high protein content, consisting mainly of animal meat. Similarly, a trans-ethnic study comparing the salivary microbiota of 52 South Koreans to that of 88 Japanese^4^ showed that the salivary microbiota of Koreans was less diverse than that of Japanese individuals. In the same study, beta diversity analyses revealed differences in community structure (weighted UniFrac) and community membership (unweighted UniFrac), in spite of close cultural, geographic and genetic relatedness. Higher abundance of 4 genera (including *Neisseria* and *Haemophilus*), and lower abundance of 17 genera (including *Prevotella*, *Veillonella*, *Fusobacterium*, *Gemella*, and *Granulicatella*) was observed in Koreans. Differences in the Korean and Japanese diet (e.g. spicy, salty, and fermented foods more prevalent in Korea) were suggested as a likely factor contributing to the observed disparities. The only study directly investigating the long-term effect of diet on the salivary microbiota compared 51 Italian vegans, 55 lacto-ovo vegetarians and 55 omnivores^5^. In spite of major differences in the macro- and micronutrient content of these diets, no difference in diversity, community structure, or taxonomic composition was observed and the authors concluded that long-term dietary habits have no effect in shaping the salivary microbiota.

Epidemiological studies have linked periodontal disease (PD) to obesity^6^, insulin resistance^7^, type 2 diabetes^8^, and cardiovascular disease^9^. A common hallmark of these disorders is systemic low-grade inflammation^10^. Periodontal disease elicits a systemic inflammatory and endotoxaemic response^11^ which suggests a direct role of the oral microbiota in cardio-metabolic pathophysiology. In a recent study of pathogen-free mice, oral administration of *Porphyromonas gingivalis*, a periodontal pathogen, induced systemic inflammation and endotoxaemia accompanied by insulin resistance, liver steatosis, and macrophage infiltration in adipose tissue, without eliciting a local inflammatory response in the periodontium^12^. Profiling of the illeal bacterial community by 16S rRNA gene sequencing showed an increased relative abundance of species-level OTUs of the order *Bacteroidales*, suggesting that the systemic effects of *P. gingivalis* were mediated by perturbation of the gut microbiota induced by inoculation of bacteria from the oral cavity. In a study of oral microbiota obtained by mouth swab from 62 patients with atherosclerosis and 30 healthy controls, Fåk and colleagues reported a weak positive correlation between genus *Parvimonas* and high sensitive C-reactive protein (CRP), thereby directly linking the oral microbiota to low-grade inflammation in cardiovascular disease.

In the present study, we sought to contribute to the ongoing elucidation of dietary effects on the oral microbial community by examining the diversity, composition and functional potential of the salivary microbiota in 78 healthy vegans and 82 healthy omnivores. We further sought to identify oral bacterial taxa associated with markers of systemic low-grade inflammation.

## Results

### Core microbiome across dietary patterns

A vegan and omnivore core saliva microbiome was defined as the genera present in >95% of omnivores and vegans, respectively. Twenty-three genera present in both core microbiomes constituted a common saliva microbiome across dietary patterns accounting for 97.0 ± 2.2% (mean ± standard deviation) of all reads in each individual (Supplementary Fig. S1A). Compositionally, the core microbiota was dominated by members of the three major phyla *Bacteroidetes*, *Firmicutes* and *Proteobacteria* (Supplementary Fig. S1B), with *Prevotella*, *Veillonella*, *Neisseria* and *Streptococcus* as the predominant genera, each with average relative abundance >5%, albeit with substantial inter-individual variation.

We identified 12 operational taxonomic units (OTU) that were present in all individuals (Supplementary Table S2). Among these were OTUs assigned to *Neisseria subflava*, *Haemophilus parainfluenzae*, *Prevotella melaninogenica*, *Veillonella dispar* and *Veillonella parvula*, as well as unclassified *Streptococcus* spp., *Granulicatella* spp. and *Campylobacter* spp. Interestingly, these 12 core OTUs accounted for more than half of all reads (51.5 ± 7.7%).

Analyses of co-occurrence and co-exclusion revealed interesting patterns among the 23 core genera (Supplementary Fig. S2). A high degree of pairwise co-occurrence was observed between *Porphyromonas* spp. and *Fusobacterium* spp. (ρ = 0.74; Q < 10^−15^) and between *Porphyromonas* spp. and *Neisseria* spp. (ρ = 0.71; Q < 10^−15^). Conversely, pronounced co-exclusion was observed between *Veillonella* spp. and *Porphyromonas* spp. (ρ = −0.73; Q < 10^−15^), *Veillonella* spp. and *Neisseria* spp. (ρ = −0.75; Q < 10^−15^), and *Neisseria* spp. and *Prevotella* spp. (ρ = −0.70; Q < 10^−15^).

### Alpha and beta diversity in vegans and omnivores

In the cohort as a whole, mean microbial richness in terms of observed and estimated (Chao1) number of OTUs was 201 ± 35.7 and 261 ± 51.0, respectively. We did not observe any difference in richness nor overall diversity as assessed by Shannon’s index and Simpson’s reciprocal index when contrasting vegans and omnivores (Supplementary Fig. S3). However, principal coordinate analysis (PCoA) revealed a subtle difference in beta-diversity in vegans and omnivores (Supplementary Fig. S4). Using permutational multivariate analysis of variance (PERMANOVA) to contrast dietary patterns, we found significant differences in community structure as assessed by Bray-Curtis (R^2^ = 2.1%, P = 0.008) and weighted UniFrac (R^2^ = 2.6%, P = 0.019) distances, but no difference for unweighted UniFrac (R^2^ = 0.8%, P = 0.125), indicating that the microbial communities in vegans and omnivores are phylogenetically similar, with difference in community structure driven by varying abundances of OTUs present in both vegans and omnivores.

### Compositional differences in vegans and omnivores

**Figure 1:**
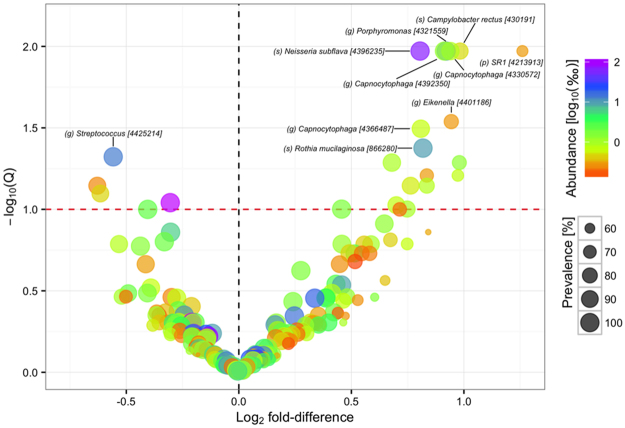
Differential abundance of operational taxonomic units. Volcano plot of estimated log_2_ fold difference in operational taxonomic unit (OTU) abundance between vegans and omnivores and corresponding Benjamini-Hochberg adjusted P-values (Q) from negative binomial Wald tests as implemented in the DESeq2 R package. The red dotted line indicates the 10% false discovery threshold. Prevalence indicates percentage of participants in which a given OTU is present. Abundance indicates mean relative abundance (‰) of a given OTU. Name of OTUs differentially abundant at an FDR ≤ 5% are given at the lowest classified rank in Greengenes [Greengenes ID]. See Supplementary Table S2 for a full list of OTUs differentially abundant at an FDR < 10%. p, phylum, o, order. f, family. g, genus. s, species.

For analysis of compositional differences between vegans and omnivores a subset of OTUs with a mean relative abundance >0.01% and prevalence >50% across diet groups were considered. Differential abundance was observed at all taxonomic levels below phylum level (Supplementary Fig. S5). A total of 22 OTUs were differentially abundant at an FDR < 10% and 10 OTUs were differentially abundant at an FDR < 5% (Fig. 1; Supplementary Table S3). Among the differentially abundant OTUs were oral and upper respiratory tract commensals like *Neisseria subflava*, *Haemophilus parainfluenzae*, *Rothia mucilaginosa*, and *Capnocytophaga* spp. which were more abundant in vegans, and *Prevotella melaninogenica* and *Streptococcus* spp. which were more abundant in omnivores. *Campylobacter rectus* and *Porphyromonas endodontalis*, species associated with periodontal disease, were also more abundant in vegans.

### Salivatypes

**Figure 2:**
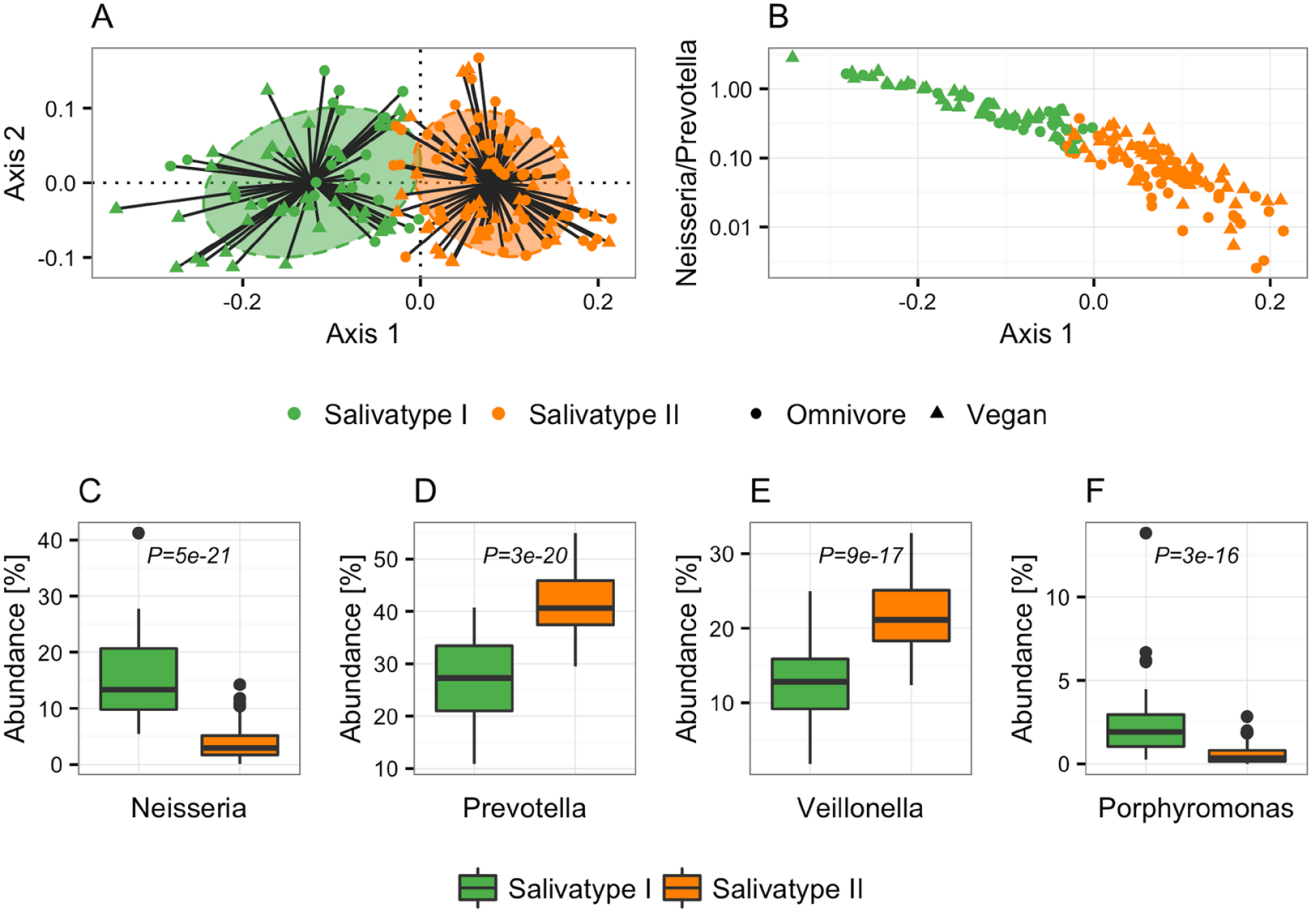
Salivatypes in vegans and omnivores. (**A**) Principal coordinates analysis visualizing salivatype clusters based on partitioning around medoids of Jensen-Shannon distance (JSD). Ellipses cover 67% samples in each cluster. (**B**) The ratio of *Neisseria* to *Prevotella* along the first principal coordinate axis of Jensen-Shannon distances used to build the salivatype clusters. (**C**–**F**) Relative abundance of the main contributors to each salivatype cluster. Differential abundance of each genus on which the clusters were build was tested using a Wilcoxon rank-sum test and the genera with P values < 10^−10^ are depicted. Boxes represent interquartile range (IQR), with the inside line representing the median. Whiskers represent values within 1.5 × IQR of the first and third quartiles. Circles represent outliers beyond the whiskers.

Clustering of samples based on genus abundance, using a partitioning around medoids (PAM) approach as previously described^13^, identified two semi-separate clusters; cluster I characterized by higher abundance of *Neisseria* and *Porphyromonas*, and cluster II characterized by higher abundance of *Prevotella* and *Veillonella* (Fig. 2). Both clusters included vegan and omnivore samples, with 46.2% of vegans and 34.1% of omnivores assigned to cluster I, and 53.8% of vegans and 65.9% of omnivores assigned to cluster II (P = 0.15, Fisher’s exact test). To address the issue of salivatypes as biological gradients rather than discrete features, we plotted the ratio of the predominant genera, *Neisseria* and *Prevotella*, against the first principal coordinate axis of the Jensen-Shannon divergence used to define the salivatype clusters, showing the discrete salivatypes to be extremes of a continuum (Fig. 2B). Interestingly, the ratio of *Neisseria* to *Prevotella* was significantly higher in vegans (P = 0.0008, Wilcoxon rank-sum test), reflecting an association between dietary patterns and the compositional features underlying genus based clusters.

### Association of nutrients with microbial composition in saliva

In order to assess the impact of collinear nutrients on the diversity of the oral microbial community, we tested the association between four measures of alpha-diversity and the first 10 principal components (PC) of the daily macro- and micronutrient intake, capturing >75% of the variation in daily nutrient intake. Using multiple regression including all 10 PCs simultaneously, we found that PC3 of the diet data was negatively associated with both Shannon’s index (0.7% decrease per unit increase in PC3; 95%CI: 1.2–0.2%; Q = 0.05) and Simpson’s reciprocal index (2.6% decrease per unit increase in PC3; 4.4–0.8%; Q = 0.09). There was no association between PC3 and neither observed nor estimated OTU count, indicating an effect of the principal component on the evenness of the community rather than the richness.

Using coinertia analysis to assess the overall congruence between variation in the salivary microbiota composition at OTU level and the variation in average daily intake of macro- and micronutrients (Supplementary Fig. S6), we found a moderate but significant correlation (RV = 0.08, P = 0.002).

**Figure 3:**
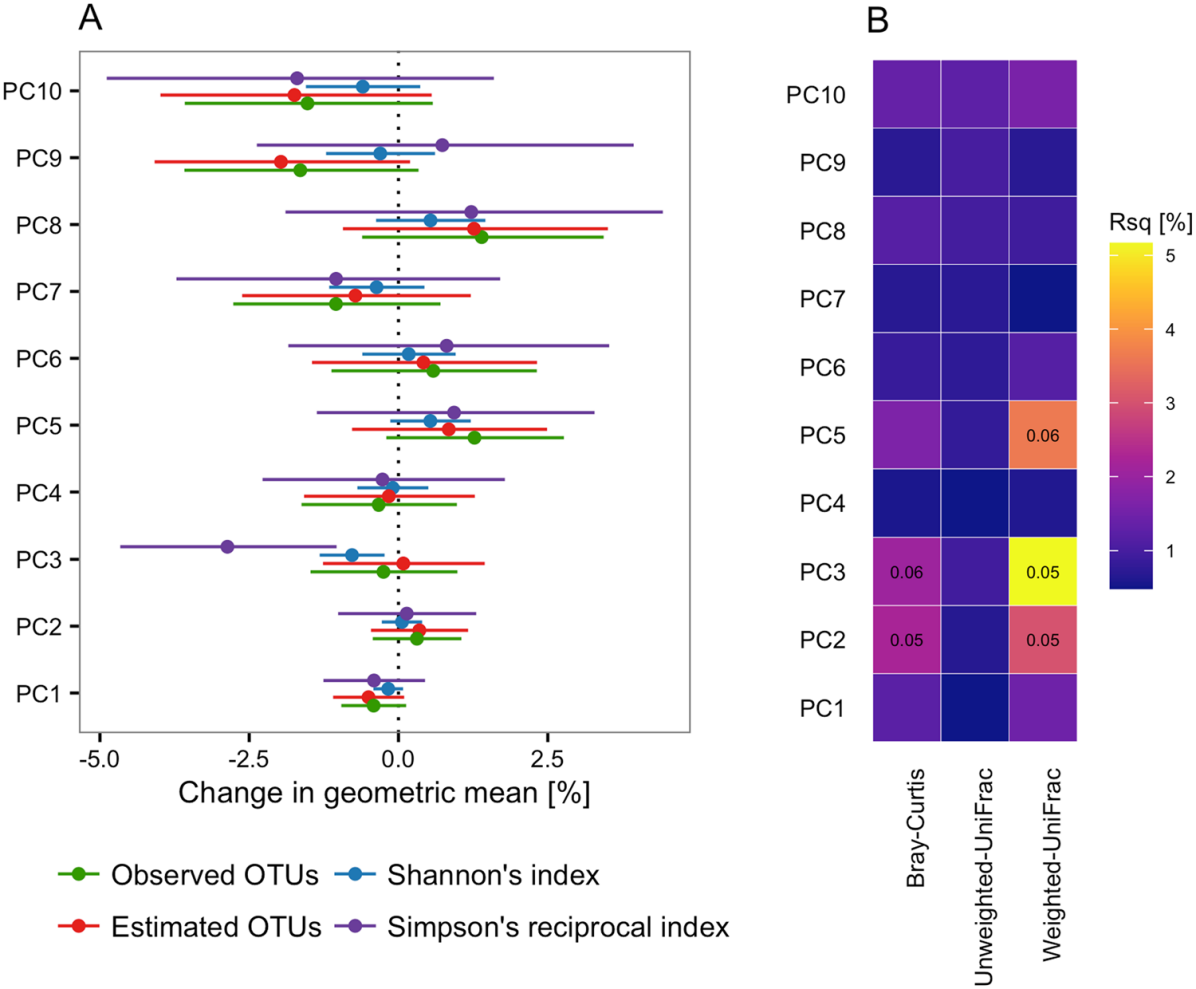
Effect of diet principal components on alpha and beta diversity. (**A**) Forest plot of effect sizes and corresponding 95% confidence intervals of the first ten diet principal components (PC) on observed richness, estimated (Chao1) richness, Shannon’s diversity index, and Simpson’s reciprocal index. Associations were tested using multiple regression including all ten PCs simultaneously. (**B**) Heatmap of variance in beta diversity explained by each of the ten first diet PCs as estimated by permutational analysis of variance. Q values are given for associations significant at an FDR ≤ 10% only.

We also tested the impact of collinear nutrients on microbial community structure and community membership using PERMANOVA, showing multivariate associations (the effect of a given PC adjusted for the remaining 9 PCs) between three diet PCs and three measures of beta diversity (Fig. 3). PC3 explained 5.2% (Q = 0.05) and 2.1% (Q = 0.06) of the variation in weighted UniFrac distance and Bray-Curtis dissimilarity, respectively. Similarly, PC2 explained 3.0% (Q = 0.05) and 2.2% (Q = 0.05) of the variation in weighted UniFrac and Bray-Curtis distances, respectively. PC5 was associated with weighted UniFrac (R^2^ = 3.6%, Q = 0.06), but not with Bray-Curtis dissimilarity. PC2 was negatively associated with dietary fibre (contributing >4% to the PC; Supplementary Fig. S7A). PC3 was negatively associated with the medium-chain fatty acids (MCFA) octanoic (caprylic, C8:0), decanoic (capric, C10:0), and dodecanoic (lauric, C12:0) acid (each contributing >7.5% to the PC; Supplementary Fig. S7B), all three constituents of coconut oil and palm kernel oil. PC5 was negatively associated with starch, and positively associated with the omega-3 polyunsaturated fatty acids (PUFA) stearidonic acid (SDA, C18:4n3), eicosapentaenoic acid (EPA, C20:5n3), docosapentaenoic acid (DPA, C22:5n3) and docosahexaenoic acid (DHA, C22:6n3) as well as the omega-9 and omega-11 mono-unsaturated fatty acids (MUFA) nervonic acid (C24:1n9) and cetoleic acid (C22:1n11), all predominantly reflecting intake of fish (Supplementary Fig. S7C). Combined, the 10 first dietary PCs explained 18.7%, 12.5%, and 8.1% of the variation in weighted UniFrac distance, Bray-Curtis dissimilarity, and unweighted UniFrac distances, respectively.

In order to clarify the compositional changes underlying the association between diet and alpha and beta diversity we focused on the subset of OTUs with a mean relative abundance >0.01% and prevalence >50% across diet groups, divided samples into quartiles according to each of PC2, PC3 and PC5, and contrasted the first and fourth quartile for each of the principal components. For each of the three PCs we found several associations significant at an FDR < 5% with substantial effect sizes ranging from two-fold to four-fold differences (Supplementary Table S4). PC2 (low fibre intake) was negatively associated with two OTUs assigned to genus *Capnocytophaga* and two OTUs assigned to genus *Neisseria*, one of which was annotated at species level as *Neisseria subflava*. Another OTU assigned to *Neisseria subflava* was negatively associated with PC5 (high piscine PUFAs and low starch). PC3 (low caprylic, capric, and lauric acid intake) was negatively associated with two OTUs assigned to genus *Prevotella*, one OTU assigned to genus *Leptotrichia* and one OTU assigned to genus *Selenomonas*. One OTU assigned to species *Veillonella dispar* was positively associated with PC2, whereas another OTU also assigned to *V. dispar* was negatively associated with PC5. Similarly, one OTU assigned to family *Neisseriaceae* was positively associated with PC3, while another OTU assigned to *Neisseriaceae* was negatively associated with PC5. PC5 was also positively associated with one OTU assigned to genus *Actinobacillus*, one assigned to genus *Lautropia*, and one assigned to genus *Porhyromonas*.

### Association of microbial composition with inflammatory biomarkers

In order to identify salivary microbiota associated with low-grade inflammation we divided samples into quartiles according to serum CRP concentration and white blood cell (WBC) count and contrasted the upper and lower quartile for each of the principal components, focusing again on the subset of OTUs with a mean relative abundance >0.01% and prevalence >50% across diet groups. We identified six OTUs associated with inflammatory makers (Supplementary Table S5); five were associated with CRP and one with WBC. One OTU assigned to *Haemophilus parainfluenzae* was associated with both CRP and WBC, but in opposite direction (positively associated with WBC and negatively associated with CRP).

### Functional differences in vegans and omnivores

Based on rarefied OTU counts the functional potential of the microbial communities in vegans and omnivores was predicted using PICRUSt^14^. A mean weighted Nearest Sequenced Taxon Index (weighted NSTI) score of 0.03 ± 0.02 indicated high reference genome coverage. Considering the resulting 6,909 KEGG orthologous groups (KOs), we did not find a difference in functional richness between vegans and omnivores. However, at an FDR < 0.10 we did find significantly higher alpha diversity indices (Shannon’s index and Simpson’s reciprocal index) in vegans compared to omnivores, indicating that the difference in functional alpha diversity is driven by difference in abundance of shared features rather than presence or absence of specific KOs. PCoA ordination of Bray-Curtis dissimilarity indicated a subtle (R^2^ = 4.0%) but significant (P = 0.002) difference in functional beta diversity (Supplementary Fig. S8). In fact, diet explained a larger proportion of the variation in the genomic potential than it did variation in community structure (4.0% vs. 2.1% variation in Bray-Curtis dissimilarity explained, respectively).

**Figure 4:**
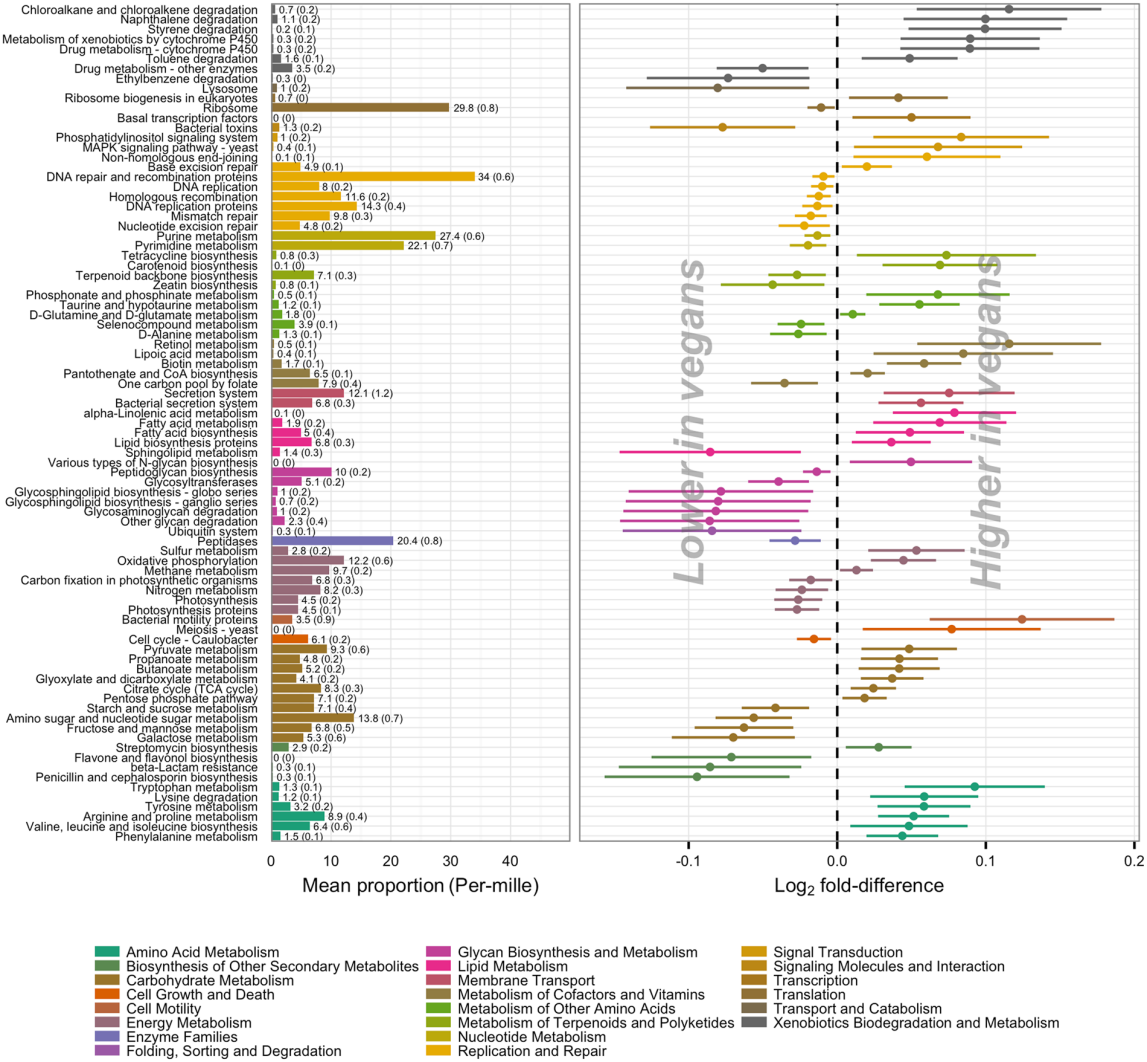
Differential abundance of KEGG pathways in vegans and omnivores. Orthologous groups were collapsed at pathway level. Pathways within the overall topics of metabolism, environmental information processing, genetic information processing and cellular processes present in ≥10% of individuals were tested for differential abundance in vegans and omnivores using the DESeq2 package. Only metabolic pathways differentially abundant at an FDR ≤ 5% are depicted. Bars indicate mean (standard deviation) proportion of differentially abundant pathways in omnivores. Forest plot indicates log_2_ fold difference and corresponding 95% confidence intervals for the difference in pathway abundance between vegans and omnivores. Pathways are coloured by functional category and ordered by decreasing log_2_ fold-difference within each category.

When collapsing KOs into KEGG pathways we identified 183 pathways within the overall topics of metabolism, environmental information processing, genetic information processing and cellular processes that were present in more than 10% of all individuals, 85 of which were differentially abundant between vegans and omnivores at an FDR < 5%; 46 pathways were enriched in vegans and 39 enriched in omnivores (Fig. 4). In general, effect estimates were small. Of the pathways enriched in vegans, 50% had a log_2_ fold-difference below 0.06, corresponding to less than a 4% increase compared to omnivores. Of the pathways enriched in omnivores, 50% had a log_2_ fold-difference above −0.04, corresponding to less than a 2.5% increase compared to vegans. Additionally, a substantial proportion of the differentially abundant pathways constituted a very low proportion of the genomic potential within each sample; 23 pathways (27%) covered less than 1‰ each.

## Discussion

In the present study of 78 vegans and 82 omnivores, we observed that the oral microbiota of vegans differed significantly from that of omnivores, both in terms of community structure and taxonomic composition, but also in terms of the genomic potential of the community. Albeit, the differences between urban dwelling vegans and omnivores in our study were small compared to the major differences observed between African agriculturalists and hunter-gatherers^3^, which at least in part are considered to reflect dietary differences. Early-life exposure as a prerequisite for diet to have a profound effect on the microbial community, or adaptation of the microbiota to dietary patterns with a loss of potential for recovery over generations, have been proposed as potential explanations to similar disparities regarding the gut microbiota^15^. Similar effects could apply to the oral microbiota, explaining why we did not observe a difference in diversity between vegans and omnivores in spite of a median 5½ year (range 1–26 years) adherence to a vegan diet; all the vegan participants in the present study changed dietary habits in their second decade of life (age at vegan debut 10–57 y), at the earliest.

Applying cluster based methods to group individuals based on the abundance of dominant genera, two partially separated clusters were identified, reflecting co-occurrence patterns of core genera, as previously described^5^. Interestingly, in terms of the optimal number of clusters and predominant genera, our results are more similar to a population-based study of middle-aged Japanese^16^ than those of a study of Italian omnivores, vegetarians and vegans^5^, perhaps reflecting that environmental factors other than diet are stronger determinants of the salivary microbiota, or simply reflecting differences in sample collection, DNA extraction, PCR amplification, and sequencing. As previously suggested regarding clustering of samples based on genus abundance in stool, the biology underlying abundance-based clustering is more likely to be gradients of co-excluding bacteria rather than discrete features^17^, which we demonstrate also applies to the oral microbiota. The fact that we observed a significant association between a vegan dietary pattern and an increased ratio of *Neisseria* to *Prevotella*, but no association between dietary patterns and the discrete salivatypes, demonstrate that quantitative analysis is generally more efficient than qualitative analysis^18^ if statistically feasible.

Our results indicate that certain dietary components influence the oral microbial community. Specifically, dietary principal components reflecting the intake of fibre, MCFAs (caprylic, capric and lauric acid), and piscine MUFAs (nervonic and cetoleic acid) and PUFAs (DHA, DPA, EPA, and SDA) were shown to be associated with diversity and community structure, driven by differentially abundant, but commonly present microbiota. The antimicrobial effects of fatty acids have been recognized for decades^19,20^. Specifically, both MCFAs, omega-3 PUFAs, and omega-9 MUFAs have been shown to have antimicrobial effects against oral microbiota *in vitro*^21–24^. However, to the best of our knowledge, this is the first report linking variation in the human salivary microbial community to fatty acids as constituents of a composite diet. In the human gut microbiota, intake of diets rich in plant-based polysaccharides have been associated with overall diversity and compositional changes at the genus level^25,26^ and non-starch polysaccharides have been experimentally demonstrated to affect the relative abundance of butyrate producing bacteria in the gut^27^. The effect of non-starch polysaccharides on the oral microbiota has not been studied with equal intensity, if at all. Interestingly, an indirect indication of an effect of dietary fibre on the oral microbial community has been provided by large-scale epidemiological studies showing an inverse relationship between dietary fibre intake and both prevalent^28^ and incident^29^ periodontal disease; an effect that appears to be preferential to cereal fibre. Our results indicate that the intake of total dietary fibre is associated with increased abundance of the oral commensals *Capnocytophaga* and *Neisseria subflava,* as well as an increase in the potential for short-chain fatty acid (butyrate and propionate) production. *Capnocytophaga* spp. are facultative anaerobic chemotrophs capable of fermenting polysaccharides including dextran, glycogen, inulin, and starch, with acetate and succinate as the major acidic end products^30^. *N. subflava* is counted among the saccharolytic *Neisseria* spp. capable of metabolizing mono- and disaccharides by oxidative processes^31^. However, further experimental studies are required to verify whether these taxa truly are responsive to the intake of dietary fibre.

Among the OTUs enriched in vegans and positively associated with CRP was one annotated as *Campylobacter rectus*, a species associated with periodontal disease^32^. The salivary microbial community mostly resembles the microbial community of the dorsum of the tongue and to what degree *C. rectus* in stimulated saliva reflects active periodontal disease has not been decisively established. A population based study of 1,198 Finnish adults indicated that, the number of different pathogenic species present in saliva is associated with clinical signs of periodontitis, rather than the presence of any single periodontal pathogen or specific combinations thereof^33^. In a study of 150 systemically healthy adults, of whom 37 were periodontally healthy and 122 had varying degrees of periodontal disease, *C. rectus* was more abundant in saliva from individuals with gingivitis or periodontitis, but receiver operator characteristic analysis showed that the presence of periodontal disease could not be reliably identified by the salivary abundance of *C. rectus*^34^. In a study of subgingival plaque from 109 individuals with periodontitis and 65 periodontally healthy controls, *C. rectus* was associated with increased CRP concentration^35^, suggesting that *C. rectus* might be a driver of the association between periodontal disease and CVD. In fact, among 1,060 individuals participating in a population-based study, the presence of *C. rectus* in subgingival plaque was associated with higher odds of recent non-fatal myocardial infarction; however, adjusting for potential confounding factors (i.e. age, sex, educational attainment, cholesterol, blood pressure, diabetes status, and smoking) abolished the association. Further suggestion of potential extra-oral effects of *C. rectus* is detection of its DNA in carotid and coronary atherosclerotic plaques^36,37^ and in amniotic fluid of preterm low birth weight pregnancies^38^. Participants in the present study were not subjected to a dental examination. Consequently, we cannot know whether the enrichment of *C. rectus* in vegan saliva reflects poor periodontal health. However, there was no difference between the vegan and omnivorous study groups with regard to demographic, anthropometric or lifestyle factors previously reported to affect the oral microbiota and the risk of periodontal disease.

Dietary patterns have been shown to have a substantial impact on the functional potential of the gut microbiota. Applying functional metagenomics, Yatsunenko and colleagues^25^ showed that the gut microbiome of high-protein consuming North-Americans was enriched in genomic potential for degradation of several amino acids, whereas the gut microbiome of low-protein consuming Malawians and Amerindians was enriched in potential for amino acid synthesis. Our results indicate that similar effects may apply to the salivary microbiota. Of notice, vegans eat significantly less protein compared to omnivores and the proportion of imputed genomic content encoding peptidase enzymes was also lower in vegans. Conversely, pathways involved in the metabolism of some of the amino acids least abundant in the vegan diet were enriched in vegans, perhaps reflecting a competitive edge for bacteria better equipped at utilizing amino acids in a habitat where this particular substrate is scarce. Similarly, the imputed potential for lipid and fatty acid biosynthesis was increased in the vegan microbiome. Increased in vegans were also the pentose phosphate pathway, pyruvate metabolism, and the potential for biosynthesis of the short-chain fatty acids butyrate and propionate. This pattern of enriched carbohydrate metabolism might reflect the higher intake of dietary fibre. The imputed potential for galactose metabolism was decreased compared to omnivores, perhaps reflecting the absence of dairy product in the vegan diet. Similarly, at the micronutrient level, the imputed genomic potential for biotin and pantothenate biosynthesis was enrich in vegans, whereas the imputed potential for folate biosynthesis was reduced, mirroring lower levels of biotin and pantothenic acid and higher levels of folate in the vegan diet. However, while these results may reflect differences in macro- and micronutrients content of the vegan and omnivorous diet, caution should be exercised in light of the small effect sizes and the relatively broad and unspecific categorization that KEGG pathways represent. In the present study, we sequenced the relatively short V4 region, which has less taxonomic resolution than longer regions like V1-V3, potentially making the functional imputation less precise. Additionally, contrary to shotgun metagenomics, bacterial 16S data can only provide indirect information about the genomic potential of a bacterial community. Although metagenomics and 16S based functional imputation are correlated across human body sites^14^ and the reference genome coverage for our samples was high, our findings require validation in other datasets, preferably applying whole metagenomics sequencing.

In summary, our study of the salivary microbiome in vegans and omnivores suggest that long-term dietary patterns and specific nutrients contribute in shaping the salivary microbiota. Our finding that certain oral bacteria are associated with circulating inflammatory markers provide further evidence to the proposed link between the oral microbiota and systemic disease.

## Methods

### Subjects and study design

Seventy-eight vegans and eighty-two omnivores were recruited through advertisements in local newspapers and online resources (Table 1). Volunteers were eligible for inclusion if they were between 18–65 years of age and weight-stable (±1 kg, assessed by interview) for a minimum of 2 months. Vegan volunteers were eligible for inclusion in the study if they had been adherent to a vegan diet for a minimum of 1 year. Volunteers who received antibiotic treatment within 3 months, had known gastrointestinal disease or reported gastrointestinal symptoms at the time of the study, or followed a medically prescribed diet were ineligible for inclusion. Pregnant and lactating women were also ineligible. The study was conducted according to the Declaration of Helsinki and was approved by the Regional Committee on Health Research Ethics for the Capital Region of Denmark (J.no. H-3-2012-145). All participants gave written informed consent.

**Table 1:** Study Sample Characteristics.

	Vegans	Omnivores	P
*Demographics*			
Participants	78 (55%)	82 (52%)	0.75^†^
Age (y)	31.2 ± 8.8	31.6 ± 8.2	0.74^‡^
*Anthropometrics*			
BMI (kg·m^−2^)	21.3 ± 2.3	21.8 ± 2.2	0.13^‡^
Body fat (%)	19.7 ± 6.0	19.5 ± 5.5	0.77^‡^
Waist circumference (cm)	77 ± 7.3	76.6 ± 7.7	0.73^‡^
*Lifestyle*			
Current smokers	8 (10%)	12 (15%)	0.48^†^
Alcohol (units·week^−1^)	2.0 (0, 6.8)	3.5 (1.0, 7.4)	0.12^$^
Physical activity (METhours·week^−1^)	38.4 ± 5.2	39.1 ± 4.6	0.34^‡^
*Socio economic status*			
Educational attainment			0.22^†^
Secondary school or less	28 (36%)	19 (24%)	0.12
Vocational training	5 (6%)	5 (6%)	>0.99
Higher education	45 (58%)	56 (70%)	0.14

Data is number of vegan and omnivorous participants (% women). Continuous traits are presented as mean ± standard deviation or median (25^th^ centile, 75^th^ centile). Discrete traits are presented as number of individuals (%). ^†^Fisher’s exact test. ^‡^Student’s T-test. ^$^Wilcoxon rank-sum test.

### Examination

Participants were examined in the morning following a 10-hour overnight fast.

#### Anthropometrics

Participants were weighed on an electronic scale (TANITA WB-110MA, Tanita Corporation of America, USA) without shoes, dressed in light clothing or underwear after having emptied their bladder. Height was measured to the nearest 0.5 cm without shoes using a wall-mounted stadiometer (ADE MZ10023, ADE, Germany). Waist circumference was measured to the nearest cm in erect position midway between the iliac crest and the lower costal margin. Body-mass index (BMI) was calculated by dividing the weight (kg) by the square of the height (m) and body fat percentage was assessed with bioelectric impedance analysis (Biodynamics BIA310e, Biodynamics corp., USA).

#### Questionnaires

All participants completed a questionnaire on lifestyle, including information on smoking and alcohol consumption. Physical activity was recorded using a validated instrument^39^, and intensity weighted activity levels were calculated as metabolic equivalents of task (MET) hours per week^40^.

#### Saliva

Participants were instructed not to brush their teeth on the morning of the examination. Saliva was collected in the fasting state, stimulated by chewing on a piece of paraffin wax. After 1 minute of mastication the participants were asked to swallow the saliva present in the mouth, after which 2 mL whole saliva, stimulated by the same piece of paraffin, was collected and immediately stored at −80 °C until analysis.

#### Blood

Blood was collected by venepuncture of the antecubital vein, fractionated, and immediately stored at −80 °C until analysis. Plasma CRP was measured on a Roche cobas c701 system using a particle-enhanced turbidimetric immunoassay (Roche Diagnostics GmbH, Mannheim, Germany) with an intra-assay CV of 0.7–2.3%. WBC was measured on an ADVIA 2120i system (Siemens Healthcare GmbH, Erlangen, Germany) using coupled flowcytometri and peroxidase methodology.

### Diet records

Diet was assessed by a self-administered 4-day weighed food record, including 2 working days and 2 weekend days within one week. Foods were quantified to the nearest 0.1 g using a calibrated precision scale (ProScale XC-2000, HBI Europe, Erkelenz, Germany). Diet was recorded validly by 149 participants. Nutrient content was calculated using the Dankost Pro software (version 1.5.49.21), which is based on the food database at the Danish Food Composition Databank containing 1,049 food items (www.frida.fooddata.dk). Vegan recipes not included in the database were constructed by qualified personnel and based on foods with complete validity in the database. Average daily intake (ADI) of macro- and micronutrients (Supplementary Table S1) was calculated as:

$${\rm{ADI}}=(({\rm{average}}\,{\rm{on}}\,{\rm{working}}\,{\rm{days}}\,\times 4)+({\rm{average}}\,{\rm{on}}\,{\rm{weekend}}\,{\rm{days}}\,\times \,3))/7$$

### DNA extraction, 16S rRNA library preparation and sequencing

Genomic DNA was isolated from 300 µL of saliva using the NucleoSpinSoil kit (Macherey-Nagel GmbH & Co. KG, Germany) following the manufacturer’s instruction. For the cell lysis, buffer SL2 + Enhancer buffer SX were used, the subsequent vortex step was replaced with repeated bead beating. DNA yield, purity and integrity were assessed using a Qubit 2.0 fluorometer, a NanoDrop 2000 spectrometer (Thermo Fisher Scientific Inc., MA USA) and agarose gel electrophoresis, respectively. Library preparation with polymerase chain reaction (PCR) amplification was performed using 20 ng bacterial DNA, 0.2 μM of each barcoded forward and reverse primer, and HotMasterMix (5Prime) solution in a total volume of 25 μL. To target the variable region 4 (V4) of the 16S rRNA gene a forward primer 515 F (5′AATGATACGGCGACCACCGAGATCTACAC <i5> TATGGTAATTGTGTGCCAGCMGCCGCGGTAA3′) and a reverse primer 806 R (5′AAGCAGAAGACGGCATACGAGAT <i7> AGTCAGTCAGCCGGACTACHVGGGTWTCTAAT3′) were used; each primer consisted of the appropriate Illumina adapter, an 8-nucleotide index sequence i5 and i7, a 10-nucleotide pad sequence, a 2-nucleotide linker, and the gene-specific primer^41,42^. The PCR reaction conditions were 3 minutes at 94 °C, followed by 28 cycles of 20 seconds at 94 °C, 30 seconds at 55 °C and 54 seconds at 72 °C on an Eppendorf thermocycler (Eppendorf AG, Germany). The samples were purified individually with a magnetic-bead based clean-up and size selection kit (Macherey-Nagel GmbH & Co. KG, Germany). Amplicons were visualized by gel electrophoresis and quantified by a Qubit 2.0 fluorometer. A master DNA pool was generated from the purified products in equimolar ratios. The DNA was sequenced using an Illumina MiSeq platform (MiSeq Reagent Kits v2, 500 cycles), generating a total of 24,663,124 (range 21,286–362,746) paired-end reads, which were merged using FLASH^43^, generating contigs comprising 250 ± 6 base pairs. Expected error filtering (Emax = 0.5) in USEARCH^44^ was used to exclude low quality contigs. Using Quantitative Insights Into Microbial Ecology (QIIME) v1.8^41^ the remaining high-quality contigs (median 47,390; inter quartile range 25,596–79,128) were de-multiplexed and assigned to operational taxonomic units by a minimum 97% sequence similarity against Greengenes v.13.8^45^ using closed reference picking. Sample coverage (Good’s estimator) was above 99% in all samples.

### Statistical analyses

All statistical tests were performed using R v3.3.1 (www.r-project.org). P-values were adjusted for multiplicity *ad modum* Benjamini-Hochberg^46^ and a false discovery rate ≤ 10% was observed for significance unless otherwise specified.

#### Microbiota composition and diversity in vegans and omnivores

Downstream analyses of 16S sequencing data were performed in R using the *phyloseq* package v1.16.2^47^. Samples were rarefied to an equal sequencing depth of 6,755 prior to alpha diversity, beta diversity and cluster analyses. Differences in richness and alpha diversity indices (Shannon’s index and Simpson’s reciprocal index) between vegans and omnivores were tested using T-tests of logarithmically transformed variables in order to improve normality and homoscedasticity. Difference in community structure between vegans and omnivores was assessed by principal coordinate ordination using Bray-Curtis, unweighted- and weighted UniFrac metrics, and tested using permutational multivariate analysis of variance as implemented in the *vegan* R package v.2.4.0. For analysis of differentially abundant taxa a negative binomial Wald test as implemented in the *DESeq2* package^48^ was applied.

Prior to cluster analysis, abundance data was de-noised by removing genera with average abundance <0.01% across all samples. Samples were clustered using partitioning around medoids (PAM) of Jensen–Shannon distances (JSD), as described by Arumugam and colleagues^13^. The optimal number of clusters was estimated to two using the Calinski–Harabasz (CH) index. The silhouette validation technique was used for assessing the robustness of clusters; mean silhouette width was 0.19 and 0.30 for clusters I and II, respectively.

#### Diet-Microbiota associations

The overall relationship between OTU-level microbiota composition and daily nutrient intake was determined by performing principal components (PC) analysis of the individual datasets followed by co-inertia analysis (as implemented in the *ade4* R package) which integrates the datasets and identifies common biological trends. The magnitude of the correlation between the datasets was quantified by the RV-coefficient (ranging from 0 to 1 with higher values indicating a higher degree of concordance) and the significance was determined by Monte Carlo simulation.

In order to assess multivariate associations between collinear nutrients and microbiome features, PC analysis of the dietary data was performed by a singular value decomposition of the centred and scaled nutrient values. The relative contribution of each nutrient to a given PC was calculated as the ratio of the loading of the nutrient to the sum of all the loadings for the PC in question. The first ten principal components, all with eigenvalues >1 and capturing 78.3% of the variance combined, were selected for analyses of association with microbiome features. In the case of alpha diversity measures, association with diet PCs was analysed using multiple linear regression with log-transformed alpha diversity measures (observed and Chao1 estimated OTU richness, Shannon’s index and Simpson’s reciprocal index) as dependent variables and PC1 through 10 as independent variables. For association with beta diversity measures (OTU based Bray-Curtis, unweighted UniFrac and weighted UniFrac distances) PERMANOVA was applied and P-values for the multivariate association of each PC was obtained by specifying a model with the PC of interest as the last in a sequence of the first ten PCs otherwise ordered by decreasing eigenvalue. To test the association of dietary PCs with individual OTUs, a negative binomial Wald test as implemented in the DESeq2 package was applied. To circumvent the assumption of constant fold change for each unit change of the independent variable, each PC was divided into quartiles and the first and fourth quartile were contrasted.

#### Functional analyses

Metagenomic prediction of 6,909 KEGG (Kyoto Encyclopaedia of Genes and Genomes) orthologous groups (KOs) was performed based on rarefied OTU counts using the default settings of PICRUSt v.0.9.1^14^. Analyses of functional alpha and beta diversity were based on the full set of imputed KOs. Difference in alpha-diversity indices between vegans and omnivores was tested using a Student’s T-test, with logarithmic transformation of the variables in order to improve normality and homoscedasticity. For functional beta-diversity analysis PCoA ordination of Bray-Curtis distances based on proportional KO abundances was performed and vegans and omnivores were contrasted using PERMANOVA. KOs were collapsed at pathway level and 120 pathways within the overall topics of metabolism, environmental information processing, genetic information processing and cellular processes with a prevalence of at least 10% were tested for differential abundance in vegans and omnivores using *DESeq2*.

### Availability of data and material

The datasets generated and/or analysed during the current study are available from the corresponding author on reasonable request.

## Electronic supplementary material

Supplementary Information
