# Supplementary material for: Impact of a vegan diet on the human salivary microbiota
Source: Sci Rep. 2018 Apr 11;8:5847. doi: 10.1038/s41598-018-24207-3 (PMC5895596; doi:10.1038/s41598-018-24207-3)
Supplement: Supplementary file 1 — Supplementary Information [file 41598_2018_24207_MOESM1_ESM.pdf]

SUPPLEMENTARY TABLES AND FIGURES

IMPACT OF A VEGAN DIET ON THE HUMAN SALIVARY MICROBIOTA

Tue H. Hansen[1], Timo Kern[1], Emilie G. Bak[1], Alireza Kashani[1], Kristine H. Allin[1], Trine Nielsen[1], Torben Hansen[1,2], Oluf Pedersen[1]

[1] The Novo Nordisk Foundation Center for Basic Metabolic Research, Section of Metabolic Genetics, Faculty of Health and Medical Sciences University of Copenhagen, Denmark

[2] Faculty of Health Sciences, University of Southern Denmark, Odense, Denmark

FIGURE S1. CORE MICROBIOTA ACROSS DIETARY PATTERNS

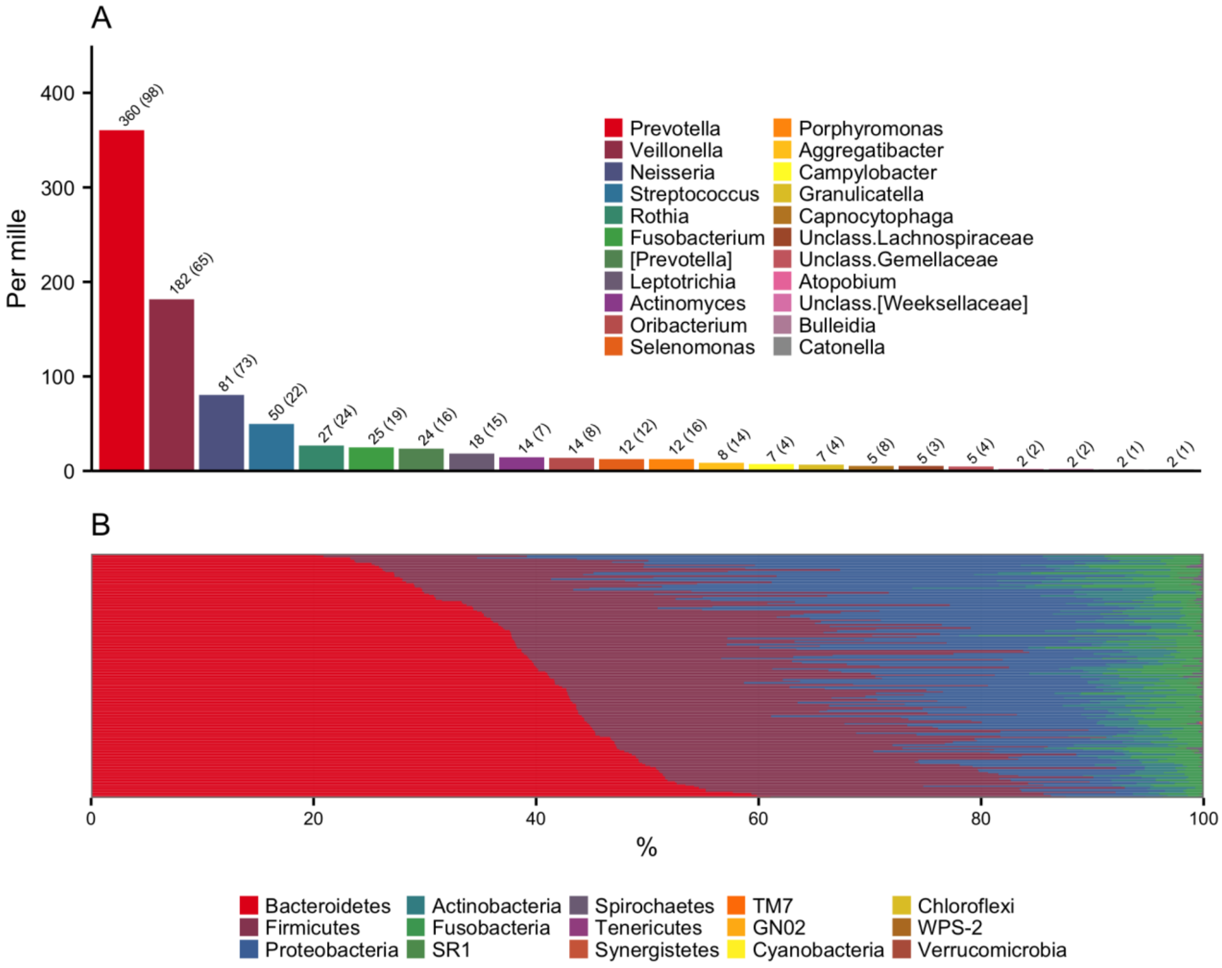

[A] Average relative abundance of 23 core genera present in >95% of individuals across dietary patterns. Mean abundance for each genus is given relative to the total number of reads per sample (‰) with standard deviation in parentheses. [B] Relative abundance of the bacterial phyla of all individual saliva samples, sorted by increasing proportion of *Bacteroidetes*.

FIGURE S2. CO-OCCURRENCE AND CO-EXCLUSION BETWEEN CORE GENERA

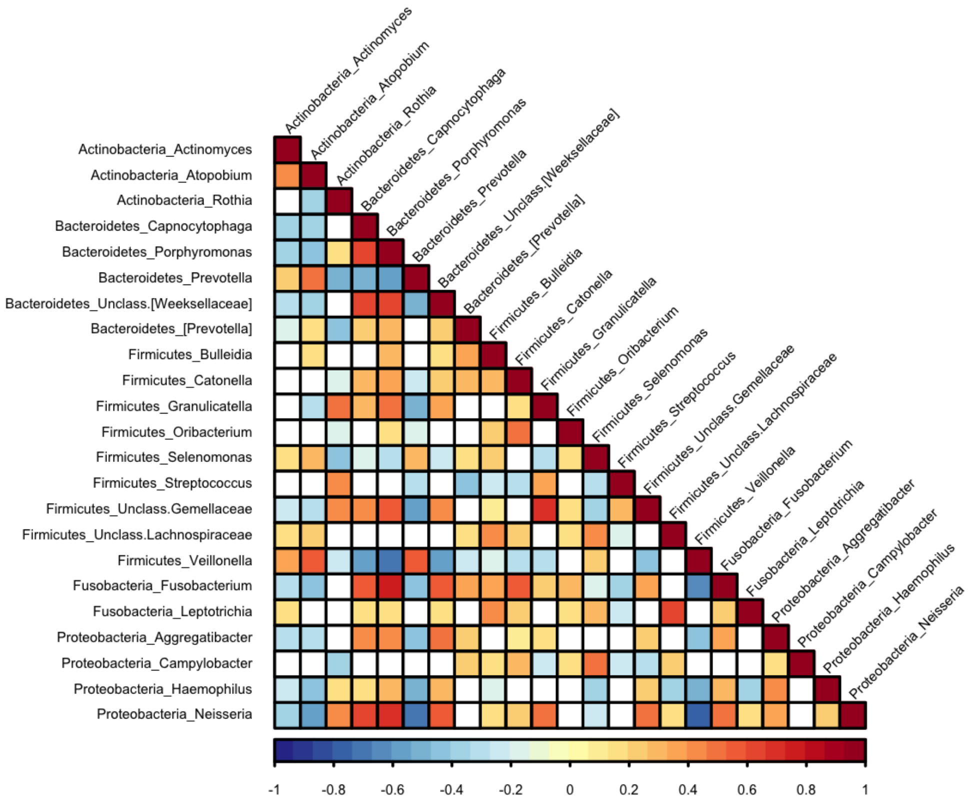

Correlogram indicating spearman correlations between the 23 core genera present in >95% of individuals across dietary patterns. For each genus the pertinent phylum is given followed by the lowest annotated taxonomic name in Greengenes (i.e. Proteobacteria_Neisseria). Genera are ordered by phylum and only correlations with false discovery rate ≤10% are shown.

FIGURE S3. ALPHA DIVERSITY IN VEGANS AND OMNIVORES

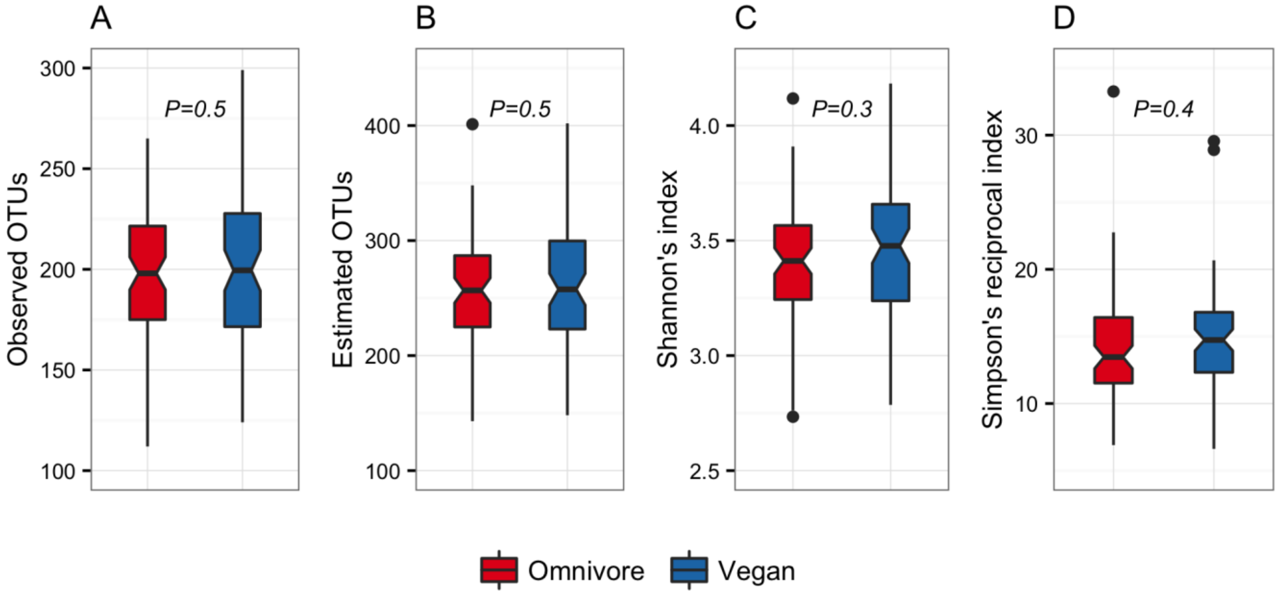

Observed richness [A], Chao1 estimated richness [B], Shannon's index [C] and Simpson's reciprocal index [D] in vegans and omnivores. Boxes represent interquartile range (IQR), with the inside horizontal line representing the median. Whiskers represent values within 1.5×IQR of the first and third quartiles. Circles represent outliers beyond the whiskers. The notch represents 50% of IQR. Difference in geometric mean was assessed by Student's t-test.

FIGURE S4. BETA-DIVERSITY IN VEGANS AND OMNIVORES

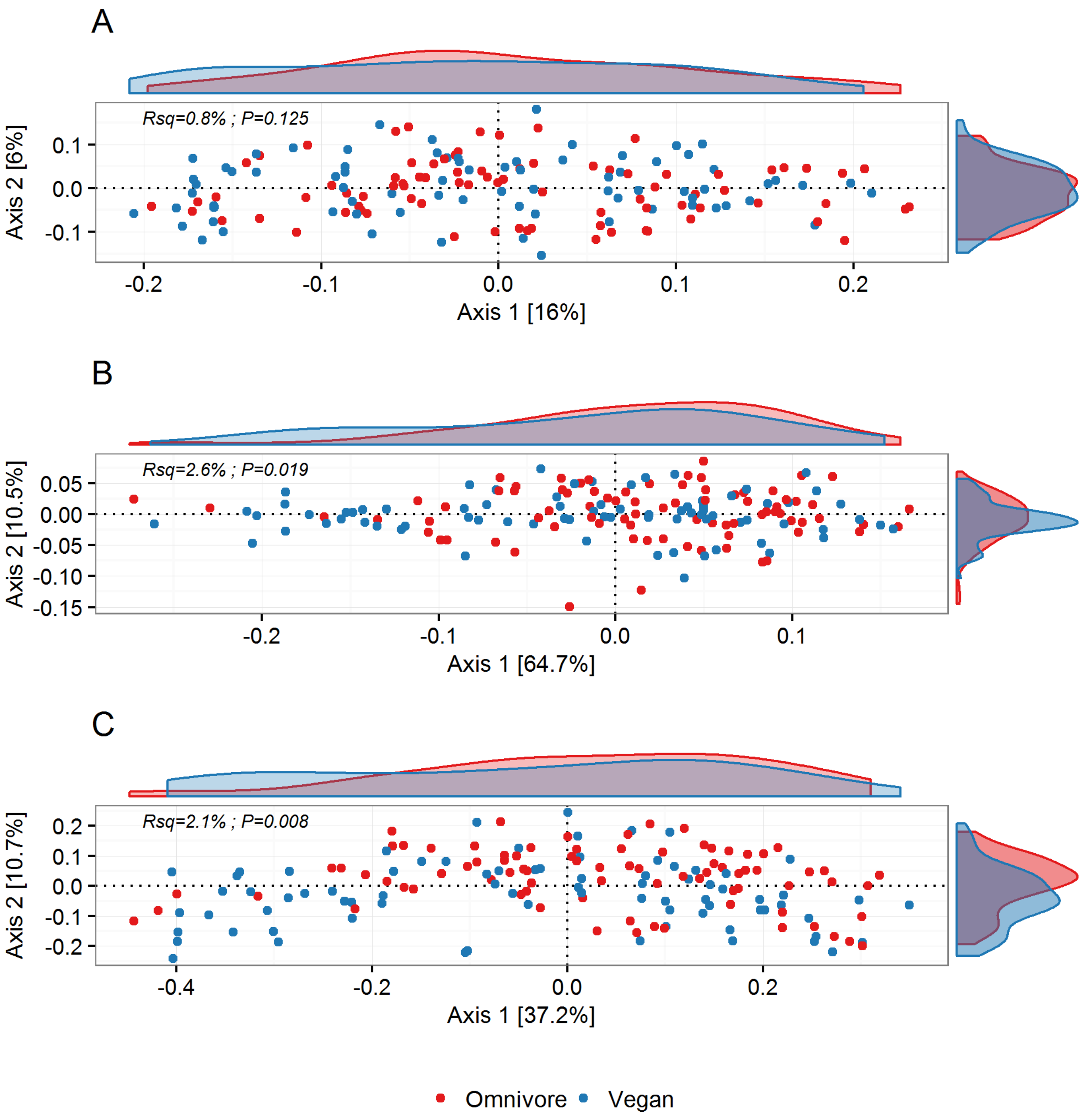

Principal coordinate ordination of microbial community structure using unweighted UniFrac [A], weighted UniFrac [B], and Bray-Curtis [C] distances. Samples are depicted in two dimensions along principal coordinate axes 1 and 2 with density along each axis. The variation in community structure explained by diet pattern (R^2 contrasting vegan vs. omnivore) was assessed by permutational analysis of variance.

FIGURE S5. DIFFERENTIAL ABUNDANCE OF HIGHER ORDER TAXA

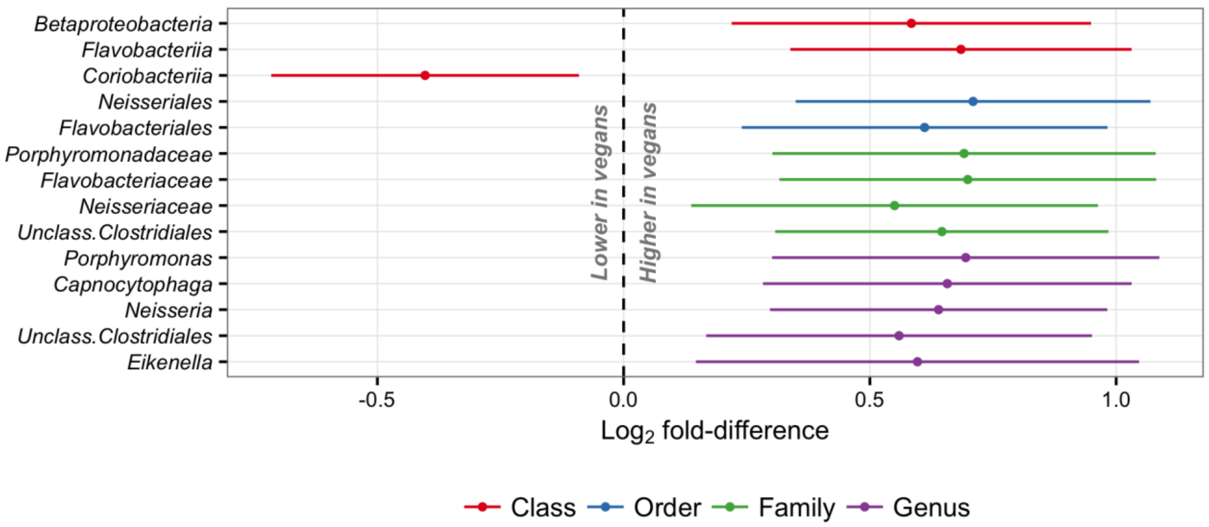

Forest plot of differential abundance of higher order taxa in vegans relative to omnivores represented by log_2 fold-difference and corresponding 95% confidence intervals from a negative binomial Wald test (DESeq2). Only taxa differentially abundant at an FDR≤10% are depicted.

FIGURE S6. COINERTIA OF SALIVARY MICROBIOTA AND DIET

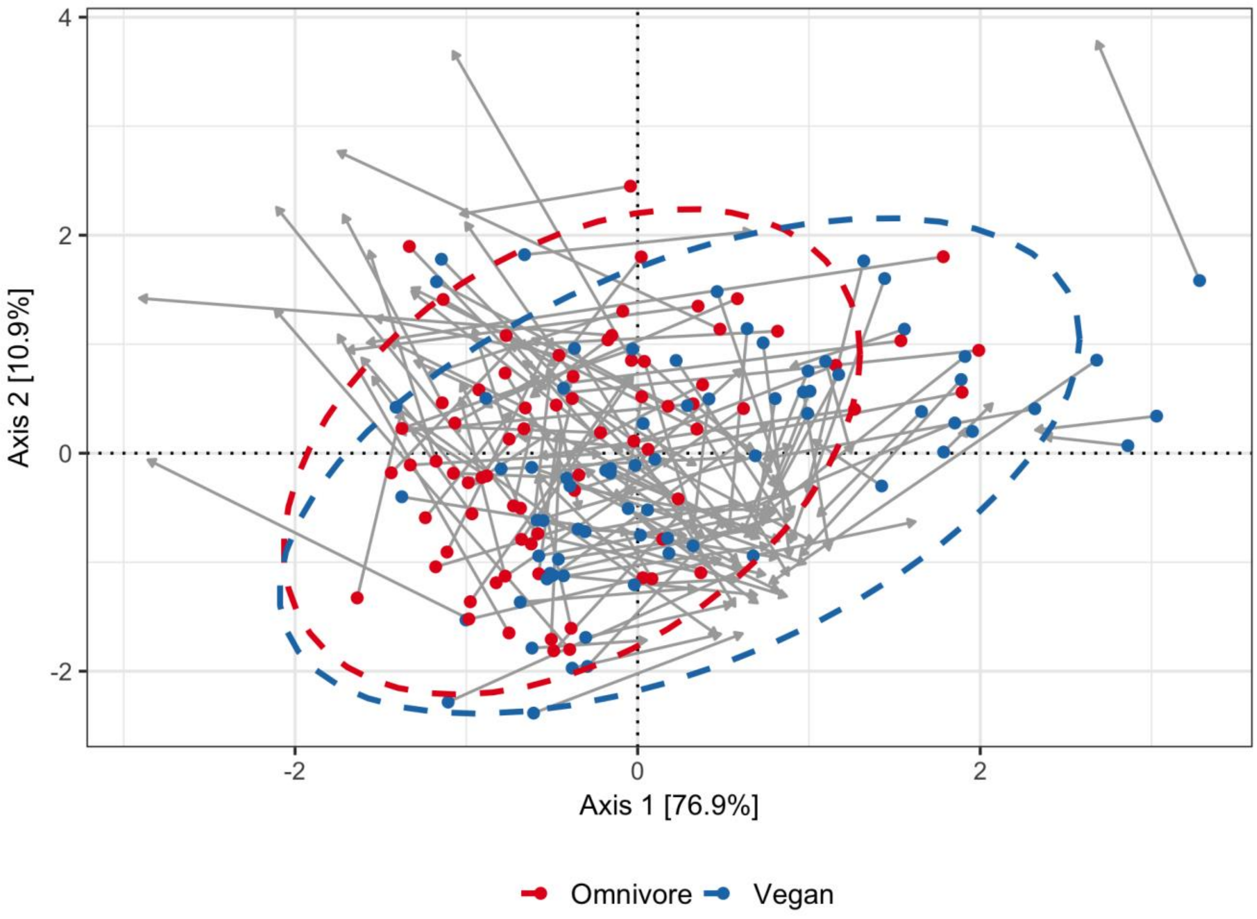

Coinertia plot depicting samples in two dimensions along coinertia axes 1 and 2. The variation captured by each axis is given in percent. Dots represent position of samples based on principal component analysis of OTU abundance and arrowheads indicate the position based on principal component analysis of the dietary data. Ellipses represent the microbiota of vegans and omnivores assuming a multivariate normal distribution with 95% confidence.

FIGURE S7. LOADING PLOTS FOR THE DIET PRINCIPAL COMPONENTS ASSOCIATED WITH ALPHA AND BETA DIVERSITY

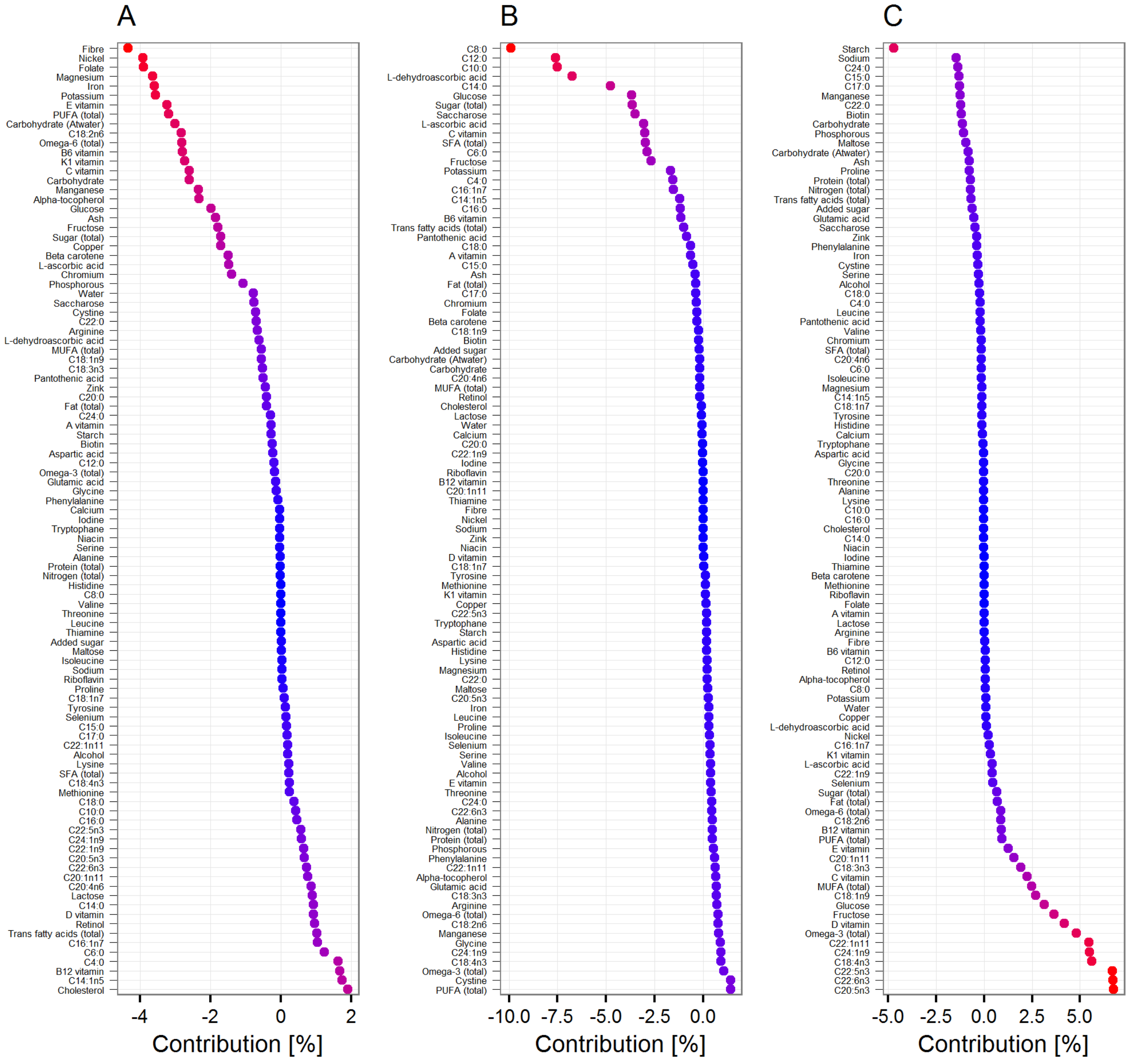

Contribution of individual nutrients to the second [A], third [B] and fifth [C] diet principal components. The relative contribution of each nutrient is calculated as the ratio of the loading of the nutrient to the sum of all the loadings for a given PC. Negative percentages indicate a negative loading of the nutrient on the PC. SFA, saturated fatty acid. MUFA, mono-unsaturated fatty acids. PUFA, poly-unsaturated fatty acids.

FIGURE S8. FUNCTIONAL ALPHA AND BETA DIVERSITY

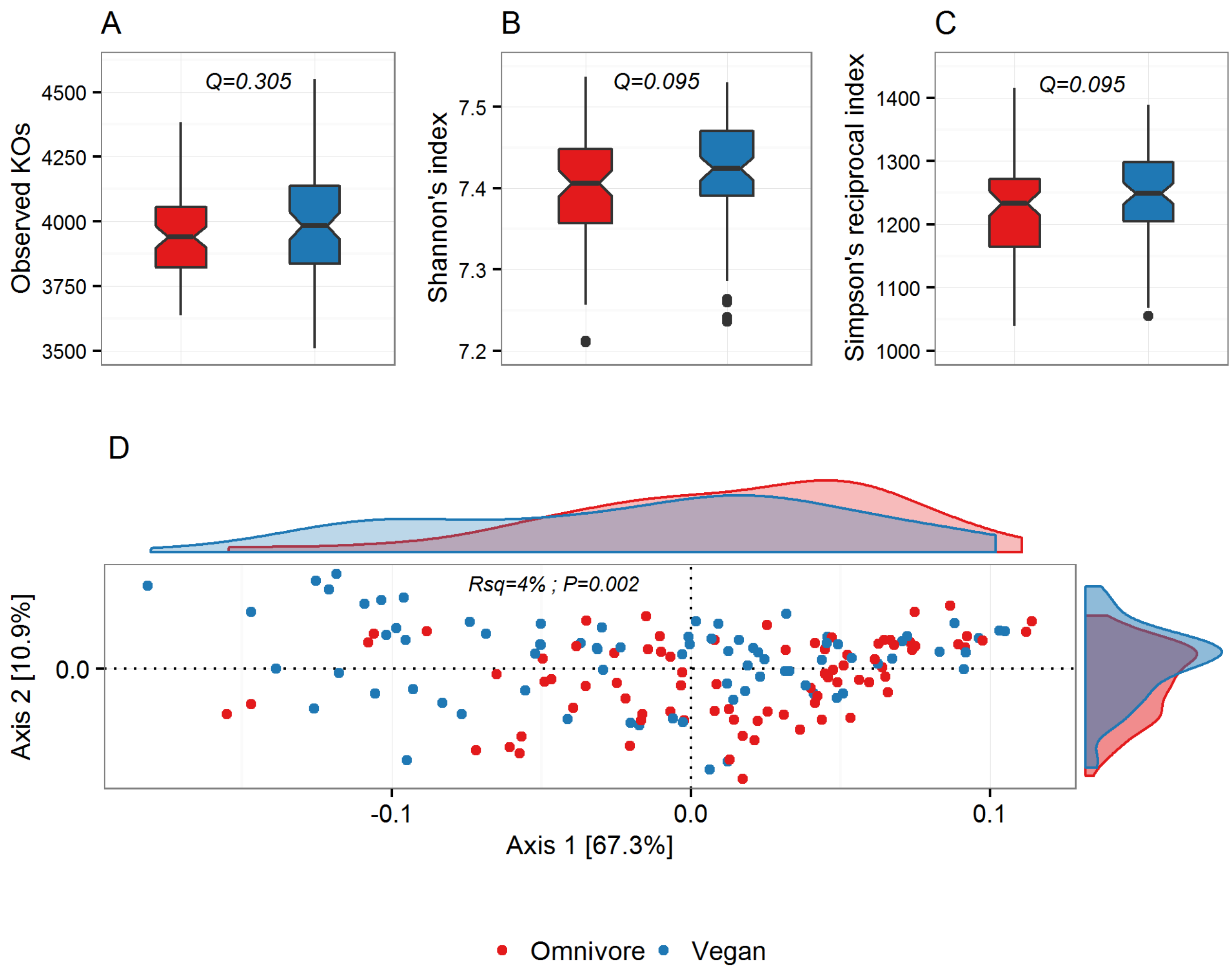

Functional alpha-diversity in vegans and omnivores as represented by observed richness [A], Shannon's index [B] and Simpson's reciprocal index [C] based on KEGG orthologs. Difference in geometric mean was assessed by t-tests. Boxplots configured similar to figure 1. [D] Principal coordinate ordination of Kyoto Encyclopaedia of Genes and Genomes orthologs (KO) using Bray-Curtis distances. Samples are depicted in two dimensions along principal coordinate axes one and two with density along each axis. The variation in functional potential explained by diet pattern (R^2 contrasting vegan vs. omnivore) was assessed by permutational analysis of variance.

TABLE S1. DAILY MACRO- AND MICRONUTRIENT INTAKE IN VEGANS AND OMNIVORES

	**Omnivores**		**Vegans**		**P**	**Q**
	Median	IQR	Median	IQR		
Added sugar (g)	10.4	9.8	8.4	9.7	1.4E-01	1.5E-01
Total carbohydrate (g)	113.8	17.5	135.8	22.1	7.3E-12	1.5E-11
Energy - carbohydrate (%)	48.9	8.1	58.5	11.6	4.0E-13	9.6E-13
Fibre (g)	12.7	4.0	19.0	5.3	7.2E-17	2.3E-16
Fructose (g)	4.7	3.2	5.7	4.7	8.6E-03	1.0E-02
Glucose (g)	4.5	2.7	5.4	4.8	2.7E-03	3.4E-03
Lactose (g)	4.6	3.9	0.02	0.07	4.7E-26	7.1E-25
Maltose (g)	0.7	0.8	0.4	0.7	2.4E-03	3.0E-03
Saccharose (g)	11.8	7.8	11.3	8.7	9.3E-01	9.3E-01
Starch (g)	37.7	17.9	48.2	23.8	6.8E-03	8.1E-03
Total sugar (g)	36.1	9.4	37.3	17.1	5.3E-01	5.5E-01
C4:0 (g)	0.3	0.3	0	0	3.5E-29	2.4E-27
C6:0 (g)	0.3	0.2	0	0	1.4E-26	3.0E-25
C8:0 (g)	0.1	0.1	0	0.04	2.1E-15	5.6E-15
C10:0 (g)	0.3	0.2	0	0.04	5.0E-20	2.4E-19
C12:0 (g)	0.4	0.3	0.08	0.4	6.2E-08	1.0E-07
C14:0 (g)	1.2	0.7	0.07	0.2	1.2E-22	7.7E-22
C14:1n5 (g)	0.1	0.07	0	0	6.8E-29	2.4E-27
C15:0 (g)	0	0	0	0	4.3E-03	5.3E-03
C16:0 (g)	6.8	1.6	2.7	1.3	3.9E-24	3.7E-23
C16:1n7 (g)	0.6	0.2	0.1	0.1	1.2E-24	1.3E-23
C17:0 (g)	0	0	0	0	8.7E-04	1.1E-03
C18:0 (g)	2.8	0.9	1.2	0.7	4.5E-23	3.2E-22
C18:1n7 (g)	0.03	0.05	0.02	0.03	1.4E-01	1.5E-01
C18:1n9 (g)	10.9	4.2	9.2	5.5	1.6E-03	2.0E-03
C18:2n6 (g)	4.0	1.7	5.8	3.8	1.5E-07	2.4E-07
C18:3n3 (g)	0.7	0.5	0.8	0.7	8.5E-01	8.6E-01
C18:4n3 (g)	0	0.02	0	0	2.2E-11	4.2E-11
C20:0 (g)	0.07	0.06	0.05	0.07	1.1E-02	1.3E-02
C20:1n11 (g)	0.3	0.1	0.06	0.06	2.6E-23	2.0E-22
C20:4n6 (g)	0.02	0.03	0	0	2.9E-20	1.6E-19
C20:5n3 (g)	0.04	0.08	0	0	1.2E-15	3.3E-15
C22:0 (g)	0.03	0.03	0.02	0.09	2.3E-01	2.5E-01
C22:1n11 (g)	0	0.05	0	0	1.6E-09	2.7E-09
C22:1n9 (g)	0.01	0.04	0	0	4.8E-10	8.6E-10
C22:5n3 (g)	0.01	0.02	0	0	3.2E-12	7.0E-12
C22:6n3 (g)	0.1	0.2	0	0	2.3E-23	1.9E-22

TABLE S1 (CONTINUED)

	Omnivores		Vegans		P	Q
	Median	IQR	Median	IQR		
C22:6n3 (g)	0.1	0.2	0	0	2.3E-23	1.9E-22
C24:0 (g)	0.02	0.04	0.01	0.03	4.5E-01	4.7E-01
C24:1n9 (g)	0.01	0.02	0	0	1.7E-12	3.9E-12
Cholesterol (mg)	117.6	72.6	0.1	0.4	4.6E-26	7.1E-25
Energy - fat (%)	31.6	7.8	27.5	11.0	2.0E-05	2.9E-05
Total fat (g)	36.6	8.9	31.7	12.8	1.6E-05	2.3E-05
MUFA (g)	12.1	4.3	10.1	6.2	1.3E-03	1.7E-03
PUFA (g)	5.5	2.5	9.3	4.5	5.7E-13	1.3E-12
SFA (g)	12.9	3.3	6.0	4.0	7.6E-21	4.3E-20
Total omega-3 (g)	0.9	0.7	0.8	0.7	9.9E-03	1.2E-02
Total omega-6 (g)	4.1	1.7	5.8	3.8	1.9E-07	2.9E-07
Total transfattyacids (g)	0.5	0.3	0	0	8.8E-26	1.2E-24
Ash (g)	8.2	1.6	8.5	2.3	3.3E-01	3.5E-01
Calcium (mg)	457.4	178.3	332.3	96.9	5.7E-10	1.0E-09
Chromium (µg)	12.7	5.3	14.8	5.8	1.3E-03	1.7E-03
Copper (mg)	1.7	1.0	2.3	1.2	7.0E-04	9.4E-04
Iodine (µg)	60.9	25.5	28.5	16.7	1.8E-15	4.8E-15
Iron (mg)	5.2	1.0	6.8	2.0	7.3E-10	1.3E-09
Magnesium (mg)	179.3	50.4	229.8	66.3	2.1E-10	3.8E-10
Manganese (mg)	2.6	1.3	3.2	1.7	4.7E-04	6.4E-04
Nickel (µg)	65.2	42.8	119.0	51.0	1.7E-11	3.3E-11
Phosphorous (mg)	656.3	165.8	586.8	178.8	9.9E-04	1.3E-03
Potassium (mg)	1399.3	425.8	1670.3	454.9	1.6E-05	2.3E-05
Selenium (µg)	20.0	8.5	11.5	3.8	8.2E-17	2.6E-16
Sodium (mg)	1103.8	408.7	764.6	388.6	1.6E-07	2.4E-07
Zink (mg)	5.3	1.1	3.9	1.5	2.1E-12	4.6E-12
Alcohol (g)	2.6	6.1	0.8	3.4	7.0E-02	7.8E-02
Energy - alcohol (%)	1.7	3.6	0.6	2.1	7.5E-02	8.3E-02
Energy (kJ)	9770.0	3570.0	10004.7	4325.4	5.2E-01	5.5E-01
Water (g)	1039.9	495.1	1090.3	544.7	5.4E-01	5.6E-01
Alanine (mg)	1279.9	388.8	768.0	261.2	1.1E-16	3.2E-16
Arginine (mg)	1529.1	496.2	1172.0	576.2	3.3E-06	5.1E-06
Aspartic acid (mg)	2438.8	619.7	1757.2	702.7	4.8E-12	1.0E-11
Cystine (mg)	266.3	99.3	221.2	119.2	6.3E-04	8.5E-04
Glutamic acid (mg)	4937.0	1435.6	3424.4	1429.2	3.0E-11	5.7E-11
Glycine (mg)	1077.3	297.8	725.3	276.2	7.5E-13	1.7E-12
Histidine (mg)	709.2	261.0	430.7	150.5	2.5E-17	8.2E-17

TABLE S1 (CONTINUED)

	Omnivores		Vegans		P	Q
	Median	IQR	Median	IQR		
Isoleucine (mg)	1276.6	463.4	706.7	265.5	4.2E-20	2.1E-19
Leucine (mg)	2099.5	743.3	1183.3	452.3	1.9E-19	8.8E-19
Lysine (mg)	1800.7	707.1	748.9	354.7	1.2E-22	7.7E-22
Methionine (mg)	603.0	225.9	271.5	113.1	1.5E-23	1.4E-22
Total nitrogen (g)	6.2	1.0	4.6	1.1	5.7E-19	2.5E-18
Phenylalanine (mg)	1230.6	429.8	827.0	339.8	4.6E-14	1.2E-13
Proline (mg)	1831.4	655.0	1090.6	429.5	2.7E-18	1.0E-17
Energy - protein (%)	15.7	2.8	12.1	2.6	7.7E-19	3.1E-18
Total protein (g)	38.8	6.5	28.9	6.9	6.1E-19	2.6E-18
Serine (mg)	1308.0	515.0	796.5	315.9	3.3E-16	9.8E-16
Threonine (mg)	999.0	355.6	578.3	216.4	6.5E-18	2.2E-17
Tryptophan (mg)	333.3	105.7	207.1	84.7	6.0E-18	2.1E-17
Tyrosine (mg)	931.6	378.5	426.5	198.3	1.9E-22	1.1E-21
Valine (mg)	1588.3	537.6	893.2	344.2	3.1E-18	1.1E-17
A vitamin (RE)	344.0	271.9	229.8	207.1	1.0E-04	1.4E-04
Alpha tocopherol (mg)	3.5	1.7	6.2	4.0	1.1E-11	2.2E-11
B12 vitamin (µg)	2.2	1.3	0	0	6.8E-29	2.4E-27
B6 vitamin (mg)	0.7	0.2	0.8	0.2	1.3E-02	1.5E-02
Beta carotene (µg)	1813.6	2693.9	2493.4	2424.3	4.5E-03	5.4E-03
Biotin (µg)	16.3	6.1	11.1	5.2	5.6E-08	9.1E-08
C vitamin (mg)	55.5	45.6	93.0	49.9	1.5E-09	2.5E-09
D vitamin (µg)	1.2	1.7	0	0	4.9E-28	1.3E-26
E vitamin (aTE)	4.0	1.8	6.9	3.9	5.5E-14	1.4E-13
Folate (µg)	159.4	53.3	239.0	92.3	1.2E-14	3.2E-14
K1 vitamin (µg)	29.0	40.8	88.4	94.3	3.7E-10	6.8E-10
L-ascorbic acid (mg)	32.0	33.8	58.8	40.2	3.5E-06	5.3E-06
L-dehydroascorbic acid (mg)	3.4	3.4	4.5	4.1	2.8E-02	3.2E-02
Niacin (NE)	12.4	2.8	8.4	2.0	1.3E-18	5.3E-18
Pantothenic acid (mg)	2.3	0.6	1.9	0.7	1.7E-08	2.9E-08
Retinol (µg)	170.4	150.5	1.1	13.3	4.2E-25	4.9E-24
Riboflavin (µg)	0.7	0.2	0.5	0.1	7.5E-16	2.1E-15
Thiamine (µg)	0.6	0.1	0.7	0.2	7.1E-05	1.0E-04

Median and inter quartile range (IQR) of macro/micro-nutrients intake per day per 1000 kcal in vegans and omnivores. P values (P) are from pairwise Wilcoxon rank-sum tests and adjusted *ad modum* Benjamini-Hochberg (Q). RE, retinol equivalents.

TABLE S2. CORE OPERATIONAL TAXONOMIC UNITS ACROSS DIETARY PATTERNS

OTU	Level	Annotation	Mean RA	Min RA	Max RA
4326219	Genus	*Campylobacter*	5.6	0.1	28.1
4396235	Species	*Neisseria subflava*	69.3	0.4	342.0
4477696	Species	*Haemophilus parainfluenzae*	94.2	9.0	260.4
4304901	Genus	*Prevotella*	24.9	1.0	93.4
4465561	Species	*Prevotella melaninogenica*	138.2	8.0	281.3
271159	Genus	*Granulicatella*	5.7	0.4	21.5
4439603	Genus	*Streptococcus*	6.2	0.6	20.7
4309301	Genus	*Streptococcus*	20.9	3.7	80.8
4446902	Family	*Gemellaceae*	4.3	0.3	25.0
3801267	Species	*Veillonella parvula*	14.6	0.1	70.5
4453501	Species	*Veillonella dispar*	102.3	1.0	248.6
4458959	Genus	*Veillonella*	28.6	2.2	126.4

Mean relative abundance (‰) of operational taxonomic units (OTU) present in all individuals. The lowest annotated taxonomic level and corresponding name is given. RA, relative abundance.

TABLE S3. DIFFERENTIALLY ABUNDANT OPERATIONAL TAXONOMIC UNITS

OTU	Level	Annotation	Log_2FD	SE	Q	Abundance
4213913	Phylum	*SR1*	1.26	0.33	0.011	0.3
4396235	Species	*Neisseria subflava*	0.81	0.22	0.011	70.8
4321559	Genus	*Porphyromonas*	0.92	0.25	0.011	10.3
4330572	Genus	*Capnocytophaga*	0.94	0.26	0.011	0.8
430191	Species	*Campylobacter rectus*	0.98	0.27	0.011	0.4
4392350	Genus	*Capnocytophaga*	0.91	0.25	0.011	1.2
4401186	Genus	*Eikenella*	0.94	0.29	0.029	0.3
4366487	Genus	*Capnocytophaga*	0.81	0.25	0.032	0.6
866280	Species	*Rothia mucilaginosa*	0.82	0.26	0.042	9.9
4425214	Genus	*Streptococcus*	-0.56	0.18	0.048	13.8
4329788	Order	*Clostridiales*	0.68	0.23	0.052	0.9
4400869	Phylum	*SR1*	0.98	0.33	0.052	1.3
978067	Genus	*Kingella*	0.84	0.29	0.062	0.3
4337383	Family	*Neisseriaceae*	0.97	0.34	0.062	0.7
526804	Genus	*Streptococcus*	-0.63	0.23	0.072	0.2
2654263	Species	*Haemophilus parainfluenzae*	0.76	0.28	0.072	0.5
4423790	Species	*Porphyromonas endodontalis*	0.84	0.31	0.072	0.8
4424239	Genus	*Streptococcus*	-0.62	0.23	0.080	0.4
4465561	Species	*Prevotella melaninogenica*	-0.30	0.12	0.091	140.8
4432435	Order	*Clostridiales*	0.70	0.27	0.094	0.6
4303688	Genus	*Lautropia*	0.75	0.29	0.100	0.9
4324467	Family	*Lachnospiraceae*	0.46	0.18	0.100	1.7

Operational taxonomic units (OTUs) differentially abundant in vegans and omnivores. The lowest annotated taxonomic level and corresponding name is given. For each differentially abundant taxon the mean relative abundance (‰) in omnivores is presented. Differential abundance was tested using a negative binomial Wald test. P-values were adjusted according to Benjamini and Hochberg. Only associations significant at a false discovery rate ≤10% (Q) are listed. OTUs are ordered by increasing Q value. Log_2 FD, fold difference in abundance in vegans relative to omnivores on the log_2 scale. SE, standard error for the log_2 fold difference.

TABLE S4. DIETARY PRINCIPAL COMPONENTS AND OPERATIONAL TAXONOMIC UNIT

OTU	Level	Annotation	Log_2FD	SE	Q	PC
43347701	Species	*Veillonella dispar*	1.89	0.43	0.00334	PC2
43664871	Genus	*Capnocytophaga*	-1.46	0.37	0.01258	PC2
43923501	Genus	*Capnocytophaga*	-1.31	0.36	0.02435	PC2
43962351	Species	*Neisseria subflava*	-1.08	0.31	0.02888	PC2
43108291	Genus	*Neisseria*	-1.56	0.47	0.04259	PC2
43215591	Genus	*Porphyromonas*	-1.13	0.36	0.06015	PC2
8662801	Species	*Rothia mucilaginosa*	-1.17	0.38	0.06502	PC2
44237901	Species	*Porphyromonas endodontalis*	-1.33	0.43	0.06731	PC2
42139131	Phylum	*SR1*	-1.41	0.47	0.07178	PC2
44002601	Genus	*Leptotrichia*	-1.39	0.46	0.07178	PC2
44095451	Genus	*Porphyromonas*	-1.18	0.39	0.07178	PC2
1094131	Genus	*Actinobacillus*	1.02	0.35	0.07593	PC2
4301911	Species	*Campylobacter rectus*	-1.12	0.39	0.08861	PC2
44807772	Genus	*Prevotella*	-1.16	0.30	0.01613	PC3
43373832	Family	*Neisseriaceae*	1.61	0.45	0.02447	PC3
44218642	Genus	*Selenomonas*	-0.99	0.28	0.02447	PC3
44410382	Genus	*Leptotrichia*	-1.44	0.41	0.02878	PC3
229512	Genus	*Prevotella*	-1.46	0.44	0.04259	PC3
8862642	Species	*Rothia mucilaginosa*	1.07	0.34	0.06015	PC3
43080982	Genus	*Leptotrichia*	-1.17	0.37	0.06015	PC3
42971192	Genus	*Leptotrichia*	-1.13	0.37	0.06731	PC3
44111382	Species	*Rothia mucilaginosa*	1.07	0.35	0.06731	PC3
43347704	Species	*Veillonella dispar*	-2.00	0.43	0.00203	PC5
43373834	Family	*Neisseriaceae*	-2.00	0.49	0.00966	PC5
1094134	Genus	*Actinobacillus*	1.43	0.39	0.02299	PC5
43962354	Species	*Neisseria subflava*	-1.12	0.31	0.02447	PC5
43036884	Genus	*Lautropia*	-1.44	0.41	0.02878	PC5
43215594	Genus	*Porphyromonas*	-1.19	0.36	0.04259	PC5
43077904	Genus	*Selenomonas*	-0.85	0.28	0.06731	PC5
8662804	Species	*Rothia mucilaginosa*	-1.11	0.38	0.08028	PC5

Operational taxonomic units (OTUs) affected by dietary principal components. The lowest annotated taxonomic level and corresponding name is given. Estimates for the association between principal components two, three, and four and OTUs are given as $\log_2$ fold difference (Log_2FD) in abundance between quartile four relative to quartile one. Differential abundance was tested using a negative binomial Wald test. P-values were adjusted (Q) according to Benjamini and Hochberg. Only associations significant at a false discovery rate <10% are listed. OTUs are ordered by increasing Q for each principal component. SE, standard error for the $\log_2$ fold difference.

TABLE S5. INFLAMMATION MARKER AND OPERATIONAL TAXONOMIC UNIT

**OTU**	**Level**	**Annotation**	**Log_2FD**	**SE**	**Q**	**Inflammation marker**
4366487	Genus	*Capnocytophaga*	1.35	0.36	0.046	CRP
430191	Species	*Campylobacter rectus*	1.25	0.38	0.062	CRP
4473129	Species	*Haemophilus parainfluenzae*	-1.25	0.37	0.062	CRP
610111	Genus	*Prevotella*	-1.42	0.44	0.069	CRP
886264	Species	*Rothia mucilaginosa*	-1.07	0.35	0.098	CRP
4473129	Species	*Haemophilus parainfluenzae*	1.28	0.36	0.084	Leukocytes

Operational taxonomic units (OTUs) associated with inflammatory markers. The lowest annotated taxonomic level and corresponding name is given. Estimates for the association between high-sensitivity C-reactive protein (CRP) or total leukocyte count (Leukocyte) and OTUs are given as $\log_2$ fold difference (Log_2FD) in abundance between quartile four relative to quartile one. Differential abundance was tested using a negative binomial Wald test. P-values were adjusted (Q) *ad modum* Benjamini-Hochberg. Only associations significant at a false discovery rate ≤10% are listed. OTUs are ordered by increasing Q for each inflammatory marker. SE, standard error for the $\log_2$ fold difference.
